# Prof. Charles A. Lee

**Published:** 1853-01

**Authors:** 


*Prof. Charles A. Lee.* — Prof. Lee has been designated, as the substitute for Dr. Smith, to pronounce the annual oration before the Alumni of Williams' College, Massachusetts, at the next annual commencement.

